# A164 SINGLE-CELL RNASEQ INDICATES INCREASE IN COLONIC REGULATORY MACROPHAGES DURING *CITROBACTER RODENTIUM* INFECTIOUS COLITIS

**DOI:** 10.1093/jcag/gwae059.164

**Published:** 2025-02-10

**Authors:** S Cortez, A Herik, R Hannawayya, A Wang, D McKay, E Cobo

**Affiliations:** University of Calgary Cumming School of Medicine, Calgary, AB, Canada; University of Calgary Cumming School of Medicine, Calgary, AB, Canada; University of Calgary, Calgary, AB, Canada; University of Calgary Cumming School of Medicine, Calgary, AB, Canada; University of Calgary Cumming School of Medicine, Calgary, AB, Canada; University of Calgary, Calgary, AB, Canada

## Abstract

**Background:**

*Citrobacter rodentium* (CR) is a murine attaching and effacing enteric bacterial pathogen that induces colitis and mimics enteropathogenic and enterohemorrhagic *E. coli* in humans. Studies of macrophages during CR infection have focused on proinflammatory functions, paying less attention to M2 or regulatory macrophages. We have shown that IL-4-treated macrophages (M(IL4)) have promising potential as a novel anti-colitic therapy using chemical-induced colitis in mice. However, any benefit of M(IL4)s in infectious colitis is unknown. This study tests the hypothesis that regulatory macrophages, specifically M(IL4)s, limit the severity of CR-infectious colitis.

**Aims:**

i. Determine if M2 macrophages increase in the colon of CR infected mice.

ii. Assess the impact of M(IL4) treatment in CR infection severity and inflammation.

**Methods:**

C57Bl/6 mice were orally infected with CR (5x10^8^ CFU), and colons were collected during the colonization, expansion, and clearance phases (0, 3, 8, 21 days post-infection, DPI) for single-cell RNAseq to examine immune profiles and differentially expressed genes (DEG). The effect of M(IL4) treatment was assessed through ip. delivery of M(IL4)s (10^6^cells) two days before CR infection followed by analysis of bacterial shedding and colonic histopathology. Inflammation was measured by neutrophil-mediated lipocalin-2 in feces by ELISA and RT-qPCR for mRNA expression of the anti-microbial peptide, REGIIIγ, and Th1 cytokine, IFN-γ.

**Results:**

Peaking at 3 DPI, there was a significant increase in arginase1^+^ Relma^+^ Ym1^+^ M2 macrophages in the colon of CR-infected mice compared to proinflammatory M1 subsets. These M2-type cells showed upregulated expression of *Cxcl9* and *Acod1* mRNA, which possess antimicrobial activity essential in mitigating CR infection. Pretreatment with M(IL4)s did not exacerbate CR infection as bacterial shedding and histopathology scores (n=13) were similar in both groups, with all mice clearing CR by 18 DPI (n=5). Infection with CR was accompanied by increased fecal lipocalin and tissue REGIIIγ and IFNγ mRNA, independent of M(IL4) treatment (n=13).

**Conclusions:**

This study suggests a formerly unappreciated antimicrobial role of M2 macrophages in the defense against CR. While M(IL4) treatment did not improve the outcome of infection with CR, neither did it exaggerate the severity of the infection, suggesting that the adoption of M(IL4) therapy for IBD would not increase the individual’s susceptibility to enteric infection.

**Funding Agencies:**

NRC

